# Expression QTL Modules as Functional Components Underlying Higher-Order Phenotypes

**DOI:** 10.1371/journal.pone.0014313

**Published:** 2010-12-13

**Authors:** Lei Bao, Xuefeng Xia, Yan Cui

**Affiliations:** 1 Department of Molecular Sciences, University of Tennessee Health Science Center, Memphis, Tennessee, United States of America; 2 Center for Integrative and Translational Genomics, University of Tennessee Health Science Center, Memphis, Tennessee, United States of America; 3 Institute of Bioinformatics, Tsinghua University, Beijing, China; National University of Ireland Galway, Ireland

## Abstract

Systems genetics studies often involve the mapping of numerous regulatory relations between genetic loci and expression traits. These regulatory relations form a bipartite network consisting of genetic loci and expression phenotypes. Modular network organizations may arise from the pleiotropic and polygenic regulation of gene expression. Here we analyzed the expression QTL (eQTL) networks derived from expression genetic data of yeast and mouse liver and found 65 and 98 modules respectively. Computer simulation result showed that such modules rarely occurred in randomized networks with the same number of nodes and edges and same degree distribution. We also found significant within-module functional coherence. The analysis of genetic overlaps and the evidences from biomedical literature have linked some eQTL modules to physiological phenotypes. Functional coherence within the eQTL modules and genetic overlaps between the modules and physiological phenotypes suggests that eQTL modules may act as functional units underlying the higher-order phenotypes.

## Introduction

Recent advances in the integration of quantitative genetics and expression genomics have provided a global view of gene expression traits and their implications in high-order phenotype variations [1], [2], [3], [4], [5], [6], [7], [8]. The Genetical Genomics [9] approach systematically associates gene expression traits with regulatory genomic regions called expression quantitative trait loci (eQTLs) [10]. Typically, this high-throughput approach identifies a large set of regulatory relations between genetic markers and expression traits, which compose bipartite networks that consist of two types of nodes, representing expression traits and eQTLs respectively.

A module is usually defined as a subset of components in a network that interact with each other and act in concert to regulate biological processes, while maintaining relative independence from other components in the network. Studies on the architecture of biological networks, including protein-protein interaction networks, metabolic networks, and transcriptional regulatory networks [11], [12], [13] have revealed that modularity is a common organizational principle of these networks. In a previous work we discovered transcription modules and their associations with higher-order phenotypes [14]. Recently a Bayesian method for eQTL network partition was developed by Zhang et al. [15]. The application of their method to a yeast eQTL network identified 20 modules with one eQTL and 9 modules with two eQTLs [15].

In this work we define eQTL module as a set of highly connected nodes with at least two eQTLs in different chromosomes. We analyzed the eQTL networks constructed from a yeast dataset and a mouse liver dataset and found 65 and 98 modules respectively. We also studied the associations between the eQTL modules and higher-order phenotypes. Genes in many eQTL modules showed significant functional coherence. Fifty yeast morphologic phenotypes were mapped to genetic loci that overlapped with the eQTLs in 19 modules. We identified an eQTL module sharing genetic components with a mouse obesity phenotype — the gonadal fat mass (GFM), and evidences from previous studies strongly support the functional relevance between the module genes and obesity. The analysis of eQTL modules may provide important insights into the functional components underlying complex phenotypes.

## Results

### Formulation of the Module Detection Problem and Simulation Results

**Figure 1:**
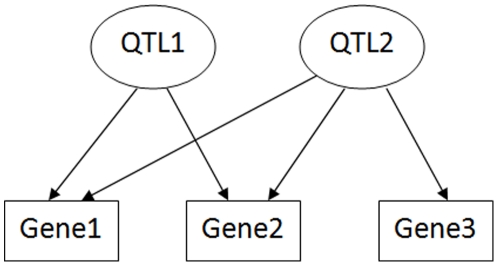
A conceptual representation of eQTL module. This module contains two eQTLs and three genes. The Q value of this module is 5/6.

We exploited a network approach to systematically analyze large numbers of modulatory relations between genetic loci and gene expression traits. A module in an eQTL network is defined as a set of highly connected nodes — eQTLs and genes whose expression levels are regulated by some or all of the eQTLs. Only eQTLs located on different chromosomes are allowed to be included in a module to avoid trivial results caused by the linkage between markers. A conceptual representation of eQTL module is shown in Figure 1. Module detection in an eQTL network can be formulated as an optimization problem: searching for a set of *m+n* nodes that maximizes the objective function *Q*(*m, n, k*) = *k*/(*m*×*n*), where *m* is the number of eQTLs, *n* is the number of target genes and *k* is the number of edges between them. In this bipartite network, genes can be connected to QTLs, but there is no edge between genes and between QTLs. The maximum number of edges between *n* genes and *m* QTLs is *m*×*n*, therefore *Q* is a value between zero and one. The objective function *Q*(*m, n, k*) is a measurement of the connection density of a module. For a set of completely connected nodes, *Q* = 1; for a set of unconnected nodes, *Q* = 0. In this work, a module must have a Q value of 0.66 or above. Intuitively, this density criterion requires that on average each gene node are connected to about 2/3 or more of the QTL nodes and *vice versa*. Besides this density criterion, a module must also be statistically significant, which means the module should be highly unlikely to arise by chance in a randomized network with the same numbers of nodes and edges and the same degree distribution. The details of the module detection method are described in Materials and Methods.

**Figure 2:**
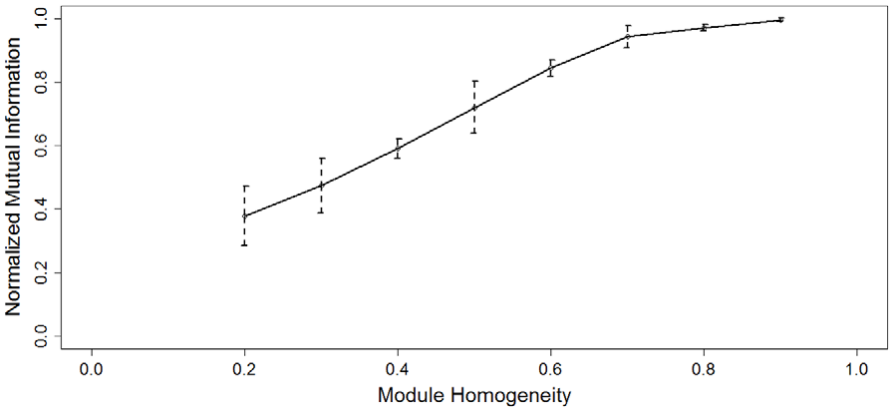
The performance of module detection algorithm as a function of module homogeneity. The error bars mark the interval of minus and plus one standard deviation.

A simulation study was performed to assess the performance of the module detection method. We generated random bipartite networks with prescribed modules and used normalized mutual information (NMI) [16] to evaluate the consistency between the prescribed modules and the modules identified by the search method. NMI is a robust performance indictor based on the confusion matrix [16]. The rows of the confusion matrix correspond to the prescribed modules, and the columns correspond to the identified modules. The confusion matrix contains the number of overlapped nodes between the prescribed modules and the identified module. If the identified modules completely match the prescribed modules, NMI takes the maximum value of 1.0; if the identified modules are unrelated to the simulated module, NMI becomes 0. The simulated eQTL networks consisted of 1200–1500 nodes and 3000–3500 edges, and contained 10 modules with 2–3 eQTL nodes and 20–150 gene nodes (typical sizes of the modules found in this work). Five independent simulation runs were performed with each of the following module homogeneity values: 0.2, 0.3, 0.4, 0.5, 0.6, 0.7, 0.8 and 0.9 (Figure 2). We then used our module detection algorithm to identify modules in the simulated networks. The details of the simulation procedure are described in Materials and Methods. The module homogeneity (*p*) controls the formation of the modular structures of the simulated network. For *p* = 1, the simulated network has a clear-cut modular structure. For *p* = 0, the prescribed modules become random partitions of the simulated network and therefore the network has no modular structure at all. The module detection algorithm is expected to be able to identify the prescribed modules correctly when *p* is high, while no module can be identified by any algorithm when *p* is too low. Our module detection method performed reasonably well with a NMI value above 0.8 when the module homogeneity was higher than 0.6, and the NMI value was very close to its maximum value of 1.0 when the module homogeneity is higher than 0.9. The NMI dropped quickly when the module homogeneity was below 0.5. This is because the modular structure became much fuzzier with such low module homogeneity values. For example, at a module homogeneity value of 0.5, on average only half of the edges connected to the nodes of a module come from members of the same module and the other half of the connections are randomly connected to nodes outside the module.

### Expression QTL Network and Modules

**Figure 3:**
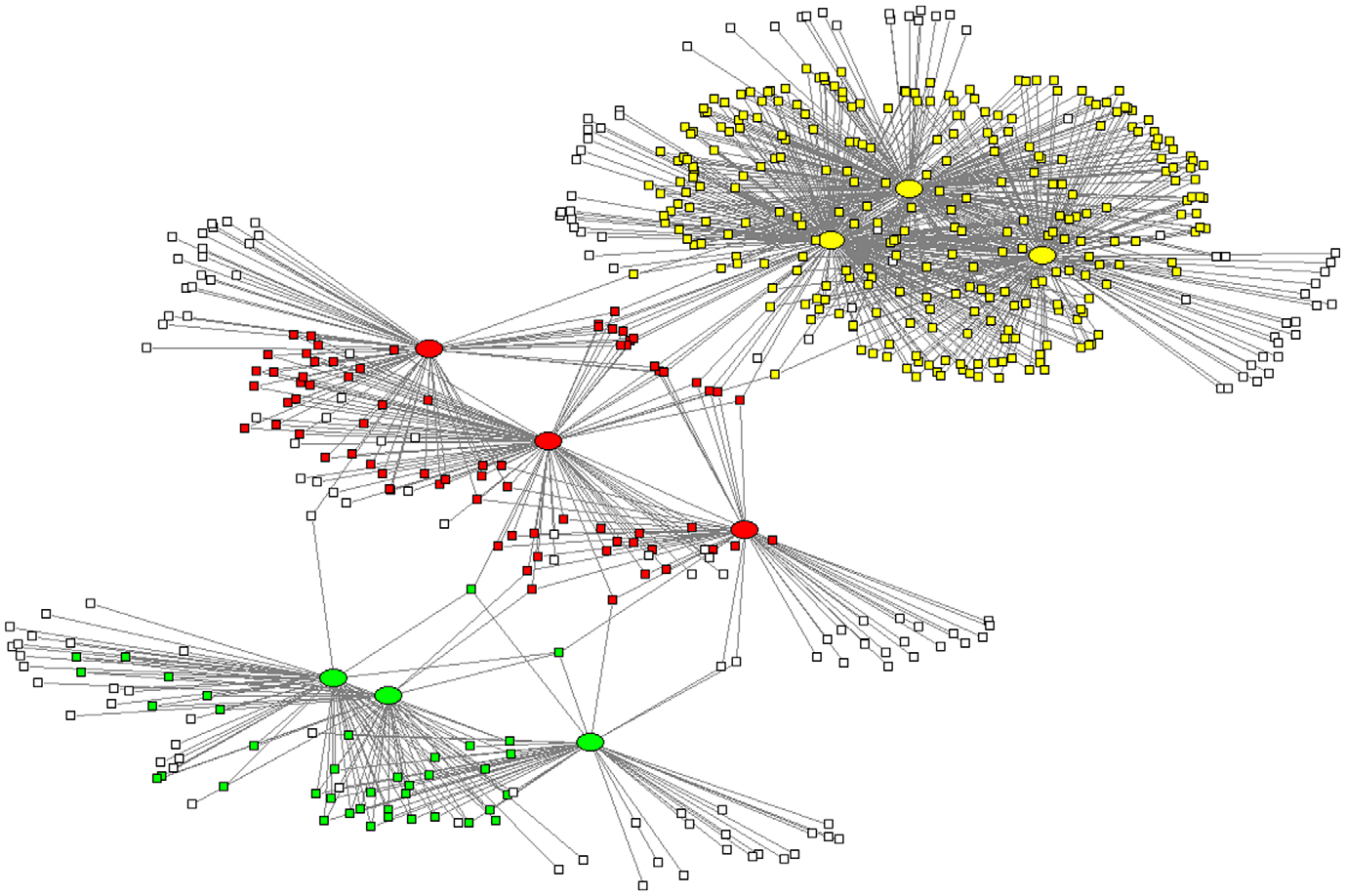
Three modules in the yeast eQTL network. The ellipses represents eQTLs, squares represent genes. White squares represent genes that do not belong to the three modules. Green: Module 48; Yellow: Module 64; Red: Module 55.

**Figure 4:**
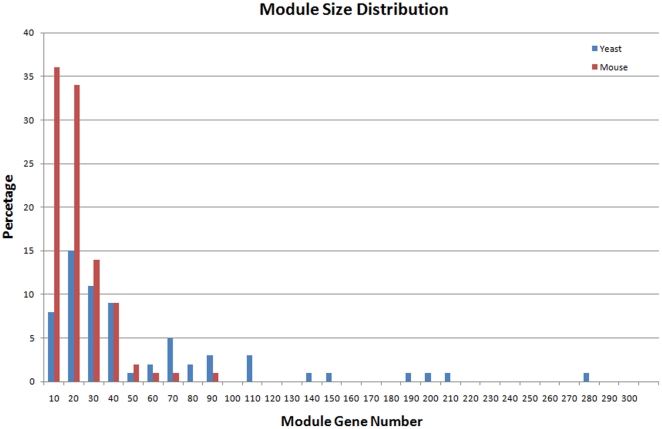
The size distributions of the yeast and mouse modules.

The yeast eQTL network is a connected graph of 493 eQTL nodes, 4583 gene nodes, and 33,584 edges. The median degrees for the eQTL nodes and gene nodes are 25 and 7 respectively. In the yeast network, we identified 65 modules (Table S1). The number of eQTLs in each module ranges from 2 to 3, and the number of target genes ranges from 4 to 276. These modules contain 1756 unique genes, covering 38.3% of the genes in the yeast eQTL network. Three identified modules and their neighboring gene nodes in the yeast eQTL network are displayed in Figure 3. The mouse liver eQTL network is a connected graph of 408 eQTL nodes, 4086 gene nodes, and 11,458 edges. The median degrees for the eQTL nodes and gene nodes are 15 and 2 respectively. In the mouse liver network, we identified 98 modules (Table S2). The number of eQTLs in each module ranges from 2 to 4, and the number of target genes ranges from 4 to 84. These modules contain 989 unique genes, covering 24.2% of the genes in the mouse eQTL network. The size distributions of the yeast and mouse modules are shown in Figure 4. We found that these modules were highly unlikely to occur simply by chance in randomly rewired networks with the same number of nodes and edges and same degree distribution (P-value <10^−4^). Therefore statistically significant modular structures exist in these eQTL networks. The modular structures of genotype-phenotype map has also been observed in some classical multiple-trait association studies [17], [18].

### Functional coherence of module genes

We used the Ontologizer software [19] to assess the enrichment of GO terms in each module. Ontologizer uses Parent-Child Analysis, which takes the structure of the GO hierarchy and parent-child relations into consideration when it performs the enrichment analysis. The Westfall-Young-Single-Step method [20] was used for multiple testing correction. A total of 42 yeast modules and 21 mouse modules were associated with at least one GO term at the significance level of P<0.05 (Tables S3 and S4).

Some modules were associated with common GO terms. For example, yeast module 63 and 64 were associated with 8 common GO terms (e.g. organelle lumen, ribosome biogenesis and assembly), and yeast module 45 and 61 were associated with 25 common GO terms (Table S3). They were identified as separate modules in the eQTL network, however there might be moderate but genuine links connecting them. These links are the weaker associations between gene expression traits of one module and eQTLs of another module, which did not pass the significance test used in eQTL mapping. We added the moderate links (with P-values <0.01 but ≥0.001) to the yeast eQTL network to test if the distribution of the moderate links would suggest potential relations between the modules. We randomly rewired the moderate links in the eQTL network and counted the number of moderate links that connected each pair of modules. For each pair of modules, the maximum number of moderate links from 1000 such randomly rewired networks was compared to the number of moderate links bridging the two modules in the original network. We then sorted the module pairs by the ratio of these two numbers (original vs. rewired maximum) in a descending order. The top 20 (1%) yeast module pairs are listed in Table S5. Among the 2080 possible yeast module pairs, modules 45 and 61 ranked 18^th^ with a ratio of 4.6, and modules 63 and 64 ranked 19^th^ with a ratio of 4.5. There were many more (4.6 and 4.5 fold respectively) moderate links bridging these module pairs in the original eQTL network than that expected by chance in the randomly rewired networks. Other top ranked module pairs that share common GO terms include: Module 46 and 64, Module 26 and 64, and Module 55 and 60. The non-random distribution of the moderate links may help us to identify modules that are more likely being functionally related.

### Linking eQTL modules to physiological phenotypes

One major goal of systems genetics is to identify gene expression modules underlying higher-order phenotypes. Recently, Nogami et al. [21] measured more than 500 yeast morphologic phenotypes and mapped 7 significant QTLs (false discovery rate [FDR] = 0.05) (Table 2 and Table S4 of [21]). We assessed the genetic overlap between these 7 morphologic QTLs and the yeast eQTL modules we identified. We found that QTLs on three chromosomes were shared by morphologic phenotypes and the modules (Table 1). The morphologic phenotypes can be classified into six categories, each representing an aspect of cellular morphology (Table 2 of [21]). Phenotypes of same category were usually mapped to QTLs on same chromosome. But there was a surprising exception where the phenotypes concerning DNA region size, position, and shape were mapped to two unlinked loci on Chromosome 14 and 15, respectively [21]. The eQTL module analysis may provide a possible explanation to the exception. The modules with eQTLs on Chromosome 14 and 15 were associated with different GO terms. Three modules (28, 45, and 61) with eQTLs on Chromosome 14 were associated with protein metabolism while three modules (7, 9, and 51) with the QTLs on Chromosome 15 were associated with mitochondrial oxidative phosphorylation and energy generation. This indicates different molecular pathways may underlie the phenotypes mapped to chromosome 14 and those mapped to chromosome 15 though they all belong to same category.

**Table 1:** Genetic overlap of yeast eQTL modules and morphologic phenotypes.

Phenotype category	QTL (bp)	Module ID
DNA region size, position and shape	chr14:440000-460000	21, 28, 50, 59, 61, 62, 63
	chr14: 480000-500000	24, 52, 58
	chr14: 500000-520000	13, 45
DNA region size, position and shape	chr15: 520000-540000	9
	chr15: 540000-560000	7, 21, 51
Mother cell size and shape	chr8: 60000-80000	56, 58
	chr8: 80000-100000	27, 37
	chr8: 100000-120000	43

**Figure 5:**
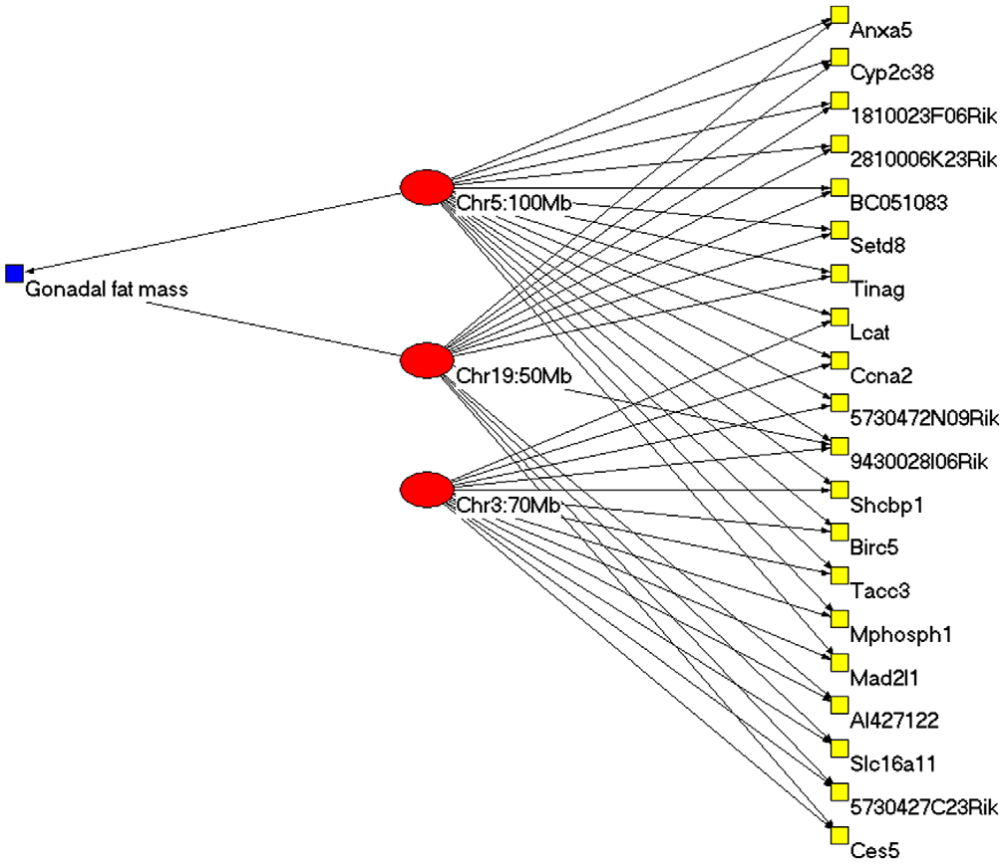
Genetic overlaps between Mouse gonadal fat mass (GFM) trait and module 74. Red ellipses represent QTLs, yellow squares represent genes and the blue square represents the GFM trait.

We also analyzed the physiological relevance of the mouse liver eQTL modules. The obesity phenotype gonadal fat mass (GFM) was genetically dissected, and five “clinical” QTLs (cQTLs) regulating this phenotype were mapped in a previous study (Table 2 of [22]). We analyzed the overlaps between the module QTLs and these five cQTLs. Three modules (50, 74, and 84) had eQTLs that overlapped with a cQTL on chromosome 19. Module 74 was of particular interest because it had another eQTL located near a cQTL on chromosome 5. The distance between the two QTL markers is about 20 Mb. This module contains three eQTLs and 21 genes, seven of which were uncharacterized expressed sequence tags (ESTs) (Figure 5). There is literature evidence for seven of the module genes (i.e. 50% of the genes in this module with known functions) being related to obesity. *Lcat* (lecithin cholesterol acyltransferase) is involved in lipid metabolism which affects the GFM trait [23]. Other module genes related to lipid metabolism and obesity include *Anxa5* (annexin A5) [24], *Ccna2* (cyclin A2) [25], *Ces5* (carboxylesterase 5) [26], *Cyp2c38* (cytochrome P450, family 2, subfamily c, polypeptide 38) [27], *Setd8* (SET domain containing 8) [28], and *Slc16a11* (monocarboxylic acid transporters, member 11) [29]. Thus, literature evidence supports the association between GFM trait and the eQTL module.

## Discussion

In this work we exploited a network approach to systematically analyze large numbers of modulatory relations between genetic loci and gene expression traits. Like many other biological networks eQTL networks have evolved functional modules. Such modular structures may confer selective advantage by allowing the optimization of gene expression within each module and therefore minimizing the impact of genetic variants outside the module.

Recently Zhang et al. [15] used a Bayesian partition method to identify eQTL modules from the same yeast dataset used in this work. They identified 20 yeast modules with one eQTL and 9 modules with two eQTLs. In this work we are interested in detecting eQTL modules with complex genetic architectures. Therefore we focused on modules with at least two QTLs in different chromosomes, and identified 21 yeast modules with two eQTLs and 44 yeast modules with three eQTLs. The Bayesian partition method [15] essentially performs eQTL mapping and module identification simultaneously. Our module detection method takes the eQTL mapping results as the input and can be used with any eQTL mapping method; therefore it provides the flexibility to reanalyze the eQTL network when new algorithms for eQTL mapping become available.

Epistasis is a higher-order genetic interaction that go beyond the pair-wise regulatory relations between a QTL and a trait. To test the epistatic effects within the eQTL modules, we employed a regression based model selection approach to find the best eQTL model for each module gene. The expression values of each module gene were regressed on the genotypes of the module eQTLs with or without interaction terms. Including interaction terms in an eQTL model may improve the model fit but also increases the model complexity. We used the standard Akaike Information Criterion to select the eQTL model with the best tradeoff between goodness-of-fit and model complexity [30]. Then for each module, we computed the proportion of module genes that could be best modeled by including the epistatic interactions. We found that this proportion ranged from 0% to 59% in the yeast modules with a median of 19%. Only four modules do not include epistatic QTLs. These results have revealed the genetic complexity of the eQTL modules.

We compared the eQTL modules of the two organisms and found that 21 (32.3%) and 44 (67.7%) yeast modules have two and three eQTLs respectively, while 12 (12.2%) and 85 (86.7%) mouse modules have two and three eQTLs respectively, and one mouse module has four eQTLs. The median module gene numbers for the yeast and the mouse modules are 27 and 14 respectively. The higher percentage of mouse modules with three eQTLs and the lower number of genes in mouse modules (Fig. 4) indicates that the regulation of gene expression in mouse is more genetically complex than that in yeast.

## Materials and Methods

### Construction of eQTL networks

We used two data sets, a yeast dataset [31], [32] and a mouse liver dataset [22], to construct the eQTL networks. The yeast data set contained genotype data of 2957 markers and gene expression data of 6216 open reading frames in 112 F1 segregants that were generated by crossing the BY4716 strain with the RM11-1a strain. Linkage analysis was performed using the Wilcoxon test, and statistical significance was estimated by permutations [31]. Significant linkage results with P-values <0.001 were used to construct the eQTL network. We divided the yeast genome into bins of 20 Kb and mapped QTLs onto them. In an eQTL network, two types of nodes were used to represent eQTLs and gene expression traits respectively, and edges represent the modulatory relations between the QTLs and gene expression traits. Gene expression traits mapped to only one eQTL were not included in the network because such nodes would not belong to any module.

The mouse liver data set contained expression data of 23,574 mouse transcripts in the livers of 334 F2 mice generated by crossing the C57BL/6J ApoE^–/–^ strain with the C3H/HeJ ApoE^–/–^ strain [22]. Using this data set, Wang et al. [22] mapped suggestive and significant eQTLs (Table S1 of [22]). Again, we used P<0.001 as the cutoff value to construct the eQTL network. We divided the mouse genome into bins of 5 Mb and mapped QTLs onto them. The QTLs modulating the GFM trait of these mice were also mapped by Wang et al. (Table 2 of [22]).

### Module detection

We employed a two-step search algorithm: in the first step we tried to find as many seed modules as possible and in the second step we merged overlapping seed modules. We searched for seed modules within a range of *m* (the number of eQTL nodes) from 2 to 6 and *n* (the number of gene nodes) from 4 to 14. For each combination of *m* and *n*, we started with a randomly picked, connected set of *m* eQTL nodes and *n* gene nodes. In each following step, one node in the current set was randomly selected and an attempt was made to replace it with a randomly picked node that does not belong to the current set but is connected to the current set by one or more edges. At the end of every 25 steps, one node in the current set was replaced with a node that had no connection to the current set to avoid getting stuck in local maxima. Changes were accepted or rejected according to the Metropolis criteria [33], [34], i.e. a move was accepted with a probability of the smaller of 1.0 or 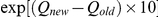 where Q_new_ and Q_old_ were the new and old Q values. The optimization continued until *Q* = 1 or 500 moves had been made. One thousand such searches with different random starts were performed and all identified seed modules (i.e., sets of connected node with a *Q* ≥0.66 and a P-value <10^−4^) were recorded. These seed modules were then merged iteratively. Each time two overlapping seed modules were merged if and only if the resulting module still had a *Q* ≥0.66 and a P-value <10^−4^. This process continued until no further merging was possible. The P-values for modules of different sizes (i.e. each combination of *m* and *n*) were estimated by random rewiring. One thousand networks were generated by randomly rewiring the edges of the original eQTL network, while keeping the edge degree of each node unchanged. The rewiring scheme is adopted from [35]. Two edges (A–B and C–D) are randomly selected and then rewired such that the new edges are A–D and B–C, provided neither of these new edges exists in the current network. This rewiring scheme is equivalent to randomly switching pairs of 0 and 1 in the rows of the adjacency matrix while keeping the raw and column margins unchanged. We then applied the module detection algorithm to these randomized networks to estimate the statistical significance of the *Q* value for each combination of *m* and *n*. One thousand independent searches with different random starts were performed for each randomized network.

### Simulation

In order to access the performance of our model detection algorithm, we generated random networks with prescribed modular structure and then used our method to identify the predefined modules. We adopted the module simulation method for bipartite network as described in [36] with minor changes to accommodate the module density criterion (*Q* value) used in this work. We first predefined the module membership for all the eQTL and gene nodes being considered. We also predefined *N_i_*, the number of gene nodes within the *i*-th module. For each eQTL node, we connected it to *N_i_* gene nodes: with probability *p*, a gene node randomly selected from the same module was connected to the eQTL; otherwise a gene node randomly selected from the whole gene node set was connected to the eQTL. The parameter *p* controls the degree of homogeneity of a module and hence is called module homogeneity. If a module generated this way did not satisfy our module density criterion (Q≥0.66), we extracted a subset of nodes from the module that met this criterion as the final module. The normalized mutual information (NMI) was used to assess the performance of the search algorithm. Given a confusion matrix in which rows are prescribed modules and columns are detected modules, NMI is defined as
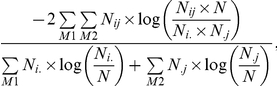
where *N_ij_* is an element of the confusion matrix specifying the number of overlapped nodes between the *i*-th prescribed module and the *j*-th detected module. *N_i._* and *N_.j_* are the row means and column means respectively, and *M_1_* and *M_2_* are the number of prescribed and detected modules [16].

## Supporting Information

Table S1 List of Yeast Modules
(0.57 MB XLS)

Table S2 List of Mouse Liver Modules
(0.19 MB XLS)

Table S3 Gene Ontology Analysis of Yeast Modules
(0.06 MB XLS)

Table S4 Gene Ontology Analysis of Mouse Liver Modules
(0.03 MB XLS)

Table S5 Yeast module pairs with significant numbers of moderate between-module links
(0.02 MB XLS)
